# Supplementary figures and images for: Similar object shape representation encoded in the inferolateral occipitotemporal cortex of sighted and early blind people
Source: PLoS Biol. 2023 Jul 25;21(7):e3001930. doi: 10.1371/journal.pbio.3001930 (PMC10368275; doi:10.1371/journal.pbio.3001930)

**A**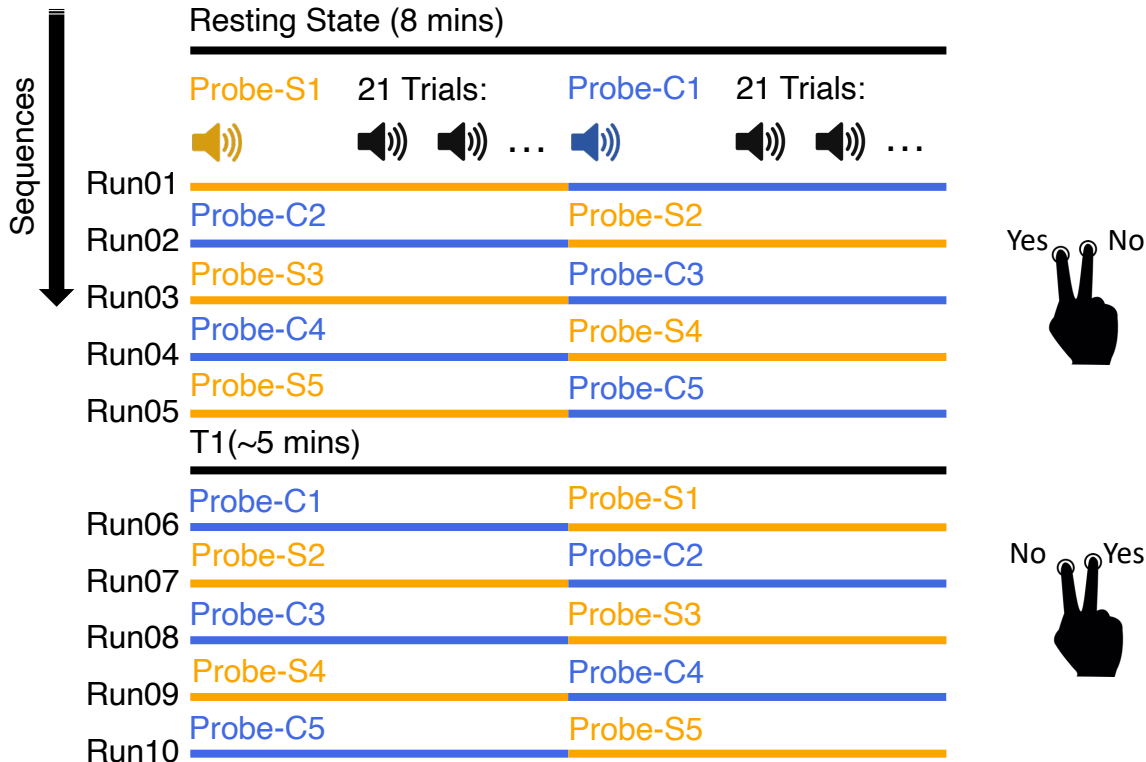**B**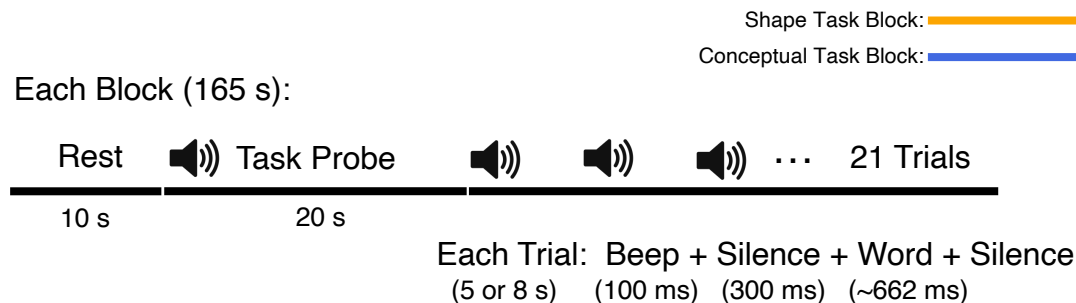

Supplement: S1 Fig — (A) The structure of the fMRI scanning session. The order of the two task blocks was interleaved across runs within each subject. Whether the first run started with a shape or a conceptual block was counterbalanced across subjects within the early blind and the sighted control group. Task probes S1 to S5 randomly corresponded to the five questions about objects’ shape for each participant (i.e., is the object elongated, angular, hollow, circular, and disc-shaped?). Task probes C1 to C5 randomly corresponded to the five questions about objects’ function for each participant (i.e., is the object used for eating, writing, sleeping, lighting, and purchasing?). The button configuration (correspondence between yes/no judgments and index/middle fingers) in the first five runs was counterbalanced across subjects within the early blind and the sighted control group. The button configuration was switched in the second set of five runs (after T1 acquisition) for each participant. (B) The timing of each block and each trial. The participants were instructed to respond by pressing buttons within 5 s. In this figure, the speaker icon is from Wikimedia Commons (https://commons.wikimedia.org/wiki/File:Speaker_Icon.svg) and the button-press icon is from Flaticon (https://www.flaticon.com/free-icon/press-with-two-fingers_4622). (PDF) [file pbio.3001930.s001.pdf]

**A**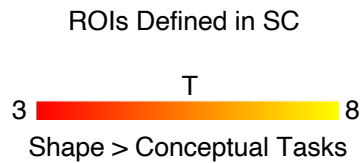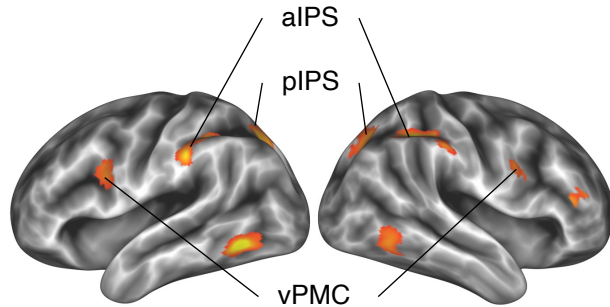**B**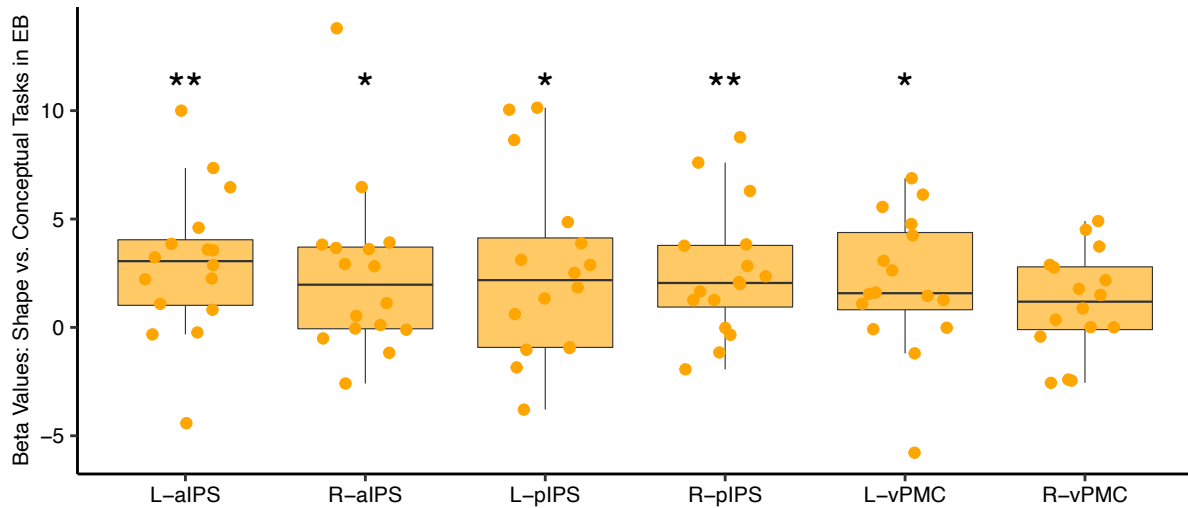

Supplement: S2 Fig — (A) ROIs defined in the contrast between shape and conceptual tasks in the sighted control (SC) (vertex-wise p < 0.001, cluster-level FWE corrected p < 0.05). (B) ROI analyses in the contrast between shape and conceptual tasks in EB using the ROIs defined in SC. *: p < 0.05, **: p < 0.01. The underlying data for this figure can be found in S1 Data. (PDF) [file pbio.3001930.s002.pdf]

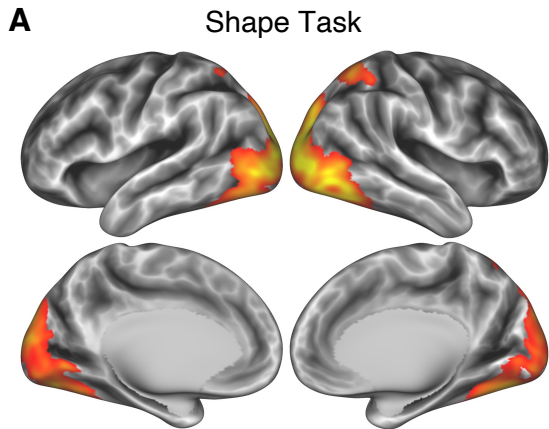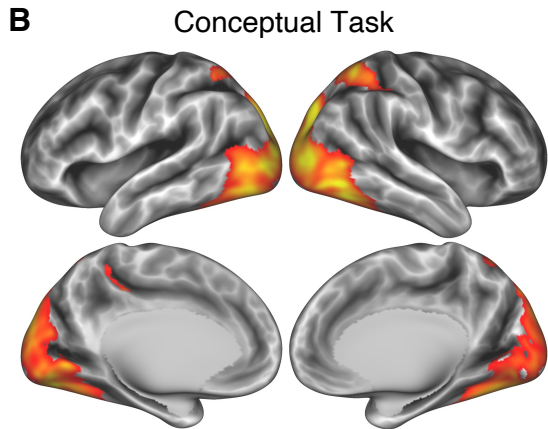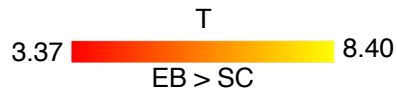

Supplement: S3 Fig — (A) EB versus SC in the shape task. (B) EB versus SC in the conceptual task. The underlying data for this figure can be found in S1 Data. (PDF) [file pbio.3001930.s003.pdf]

**A**

RT (N = 48)

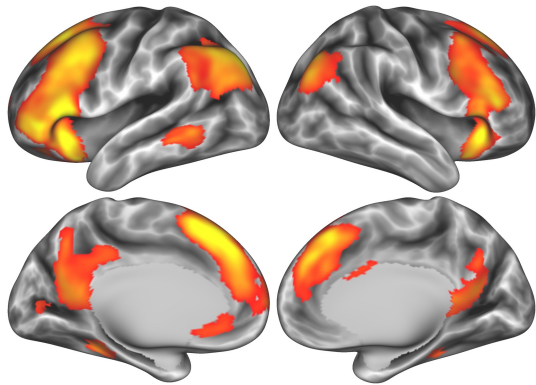**B**

RT: EB &gt; SC

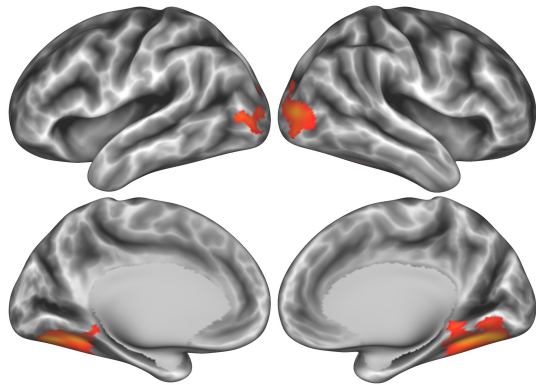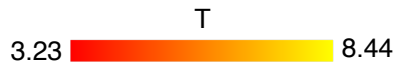

Supplement: S4 Fig — (A) The RT effect across all the participants (N = 48). (B) The differences in the RT effect between the early blind (EB) and the sighted control (SC). The underlying data for this figure can be found in S1 Data. (PDF) [file pbio.3001930.s004.pdf]

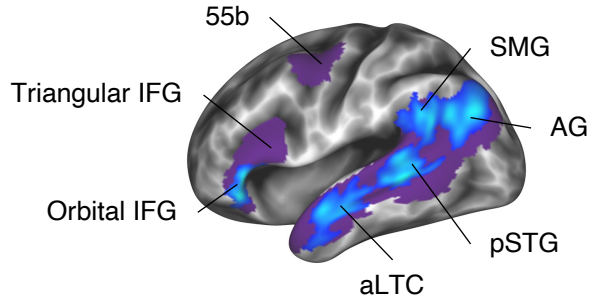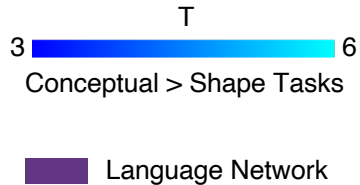

Supplement: S5 Fig — The conceptual task in our study involved a brain network (in blue) almost identical to the language network (in purple), except for the triangular part of the IFG and the 55b area in the premotor cortex. These two dorsal regions are considered to play a non-semantic role in language processing. The conceptual network (in blue) was defined in the contrast between the conceptual task and the shape task with the control of other object properties (N = 48; vertex-wise p < 0.001, cluster-level FWE corrected p < 0.05). The language network (in purple) was defined in the study by Fedorenko and colleagues [48] with the data updated from 220 participants. The overlap coefficient between these two networks was 83.05%. Such highly overlapped results suggest that the language system plays a crucial role in our conceptual task. (PDF) [file pbio.3001930.s005.pdf]

**A**

Word Duration (N = 48)

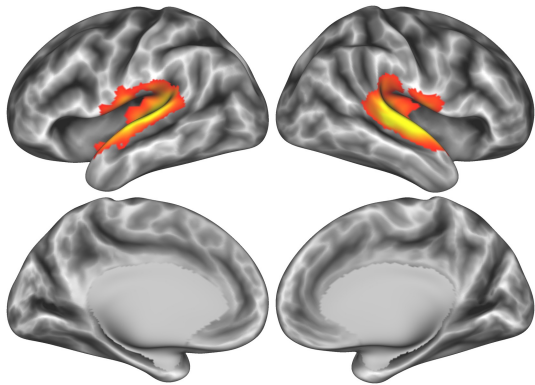**B**

Word Frequency (N = 48)

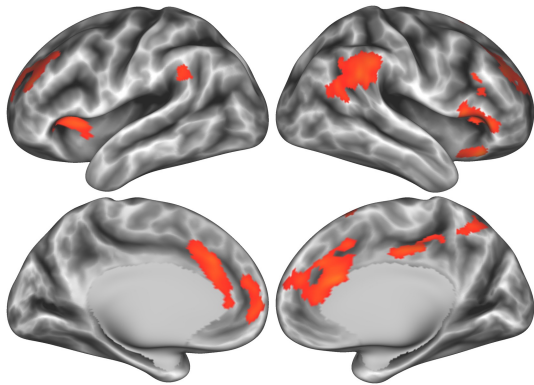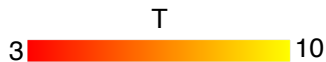

Supplement: S6 Fig — (A) Neural correlates of word duration. (B) Neural correlates of word frequency. The underlying data for this figure can be found in S1 Data. (PDF) [file pbio.3001930.s006.pdf]

**A**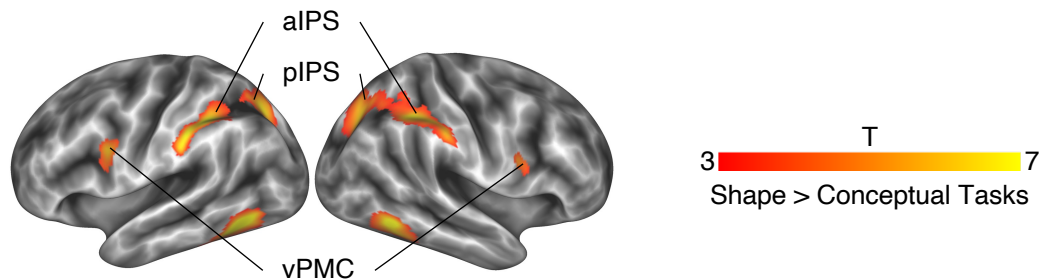**B**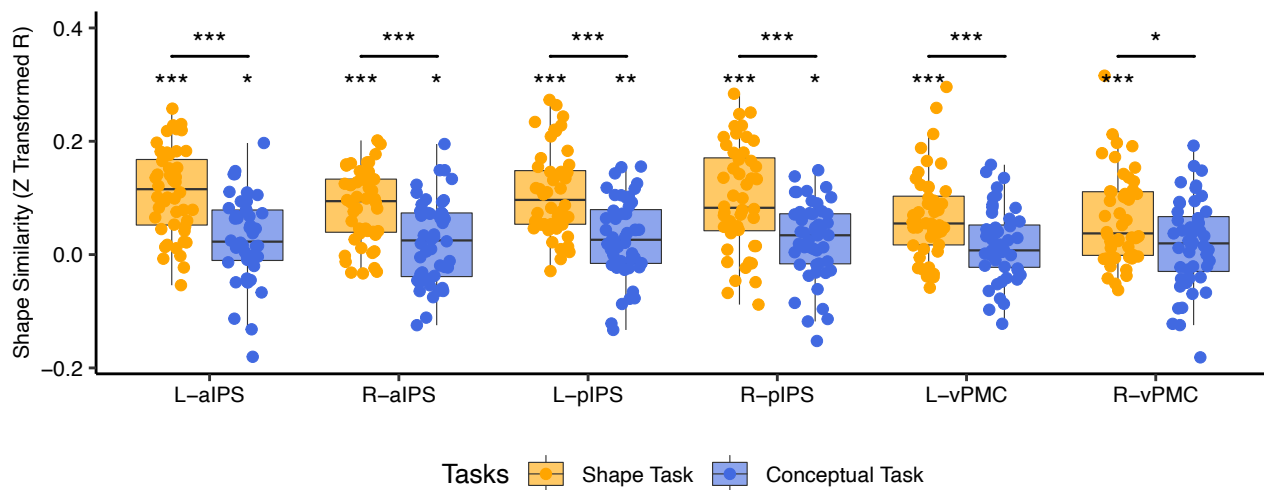

Supplement: S7 Fig — (A) Brain areas with significantly greater activation in the shape task than in the conceptual task defined in Fig 2A. (B) RSA results of these shape-relevant areas in shape and conceptual tasks. *: p < 0.05, **: p < 0.01, ***: p < = 0.001. The underlying data for this figure can be found in S1 Data. (PDF) [file pbio.3001930.s007.pdf]

**A** Shape Similarity: All (N = 48)

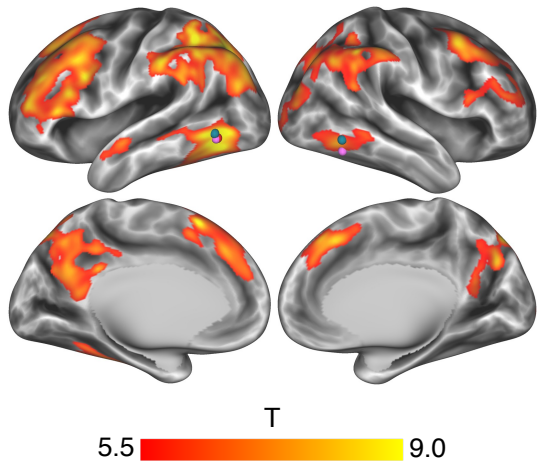

**B** Shape Similarity: EB > SC

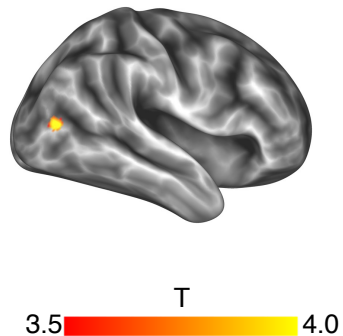

Supplement: S8 Fig — (A) Whole-brain searchlight results of shape similarity across all participants (N = 48; vertex-wise FWE corrected p < 0.005, cluster size > 400 mm2). (B) Group difference of whole-brain searchlight of shape similarity between the early blind (EB) and the sighted control (SC) (vertex-wise p < 0.001, cluster-level FWE corrected p < 0.05). The underlying data for this figure can be found in S1 Data. (PDF) [file pbio.3001930.s008.pdf]

# Conceptual Association: All (N = 48)

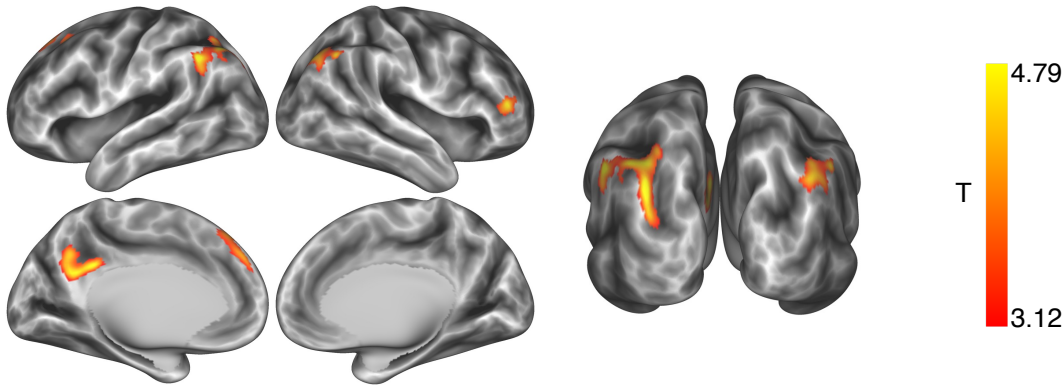

Supplement: S9 Fig — The underlying data for this figure can be found in S1 Data. (PDF) [file pbio.3001930.s009.pdf]

# RSFC of Left ILOTC: EB > SC

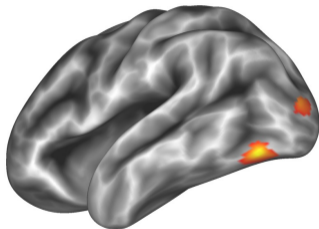

T

3.44

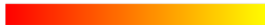

6.21

Supplement: S10 Fig — The underlying data for this figure can be found in S1 Data. (PDF) [file pbio.3001930.s010.pdf]
