# Supplementary material for: Similar object shape representation encoded in the inferolateral occipitotemporal cortex of sighted and early blind people
Source: PLoS Biol. 2023 Jul 25;21(7):e3001930. doi: 10.1371/journal.pbio.3001930 (PMC10368275; doi:10.1371/journal.pbio.3001930)
Supplement: S1 Table — (PDF) [file pbio.3001930.s011.pdf]

S1 Table. Demographic information of the early blind and their matched sighted control

Early Blind	Gender	Age	Handedness [a]	Onset of Blindness	Etiology	Sighted Control	Gender	Age	Handedness [a]
EB01	F	33	90	Birth	Retinopathy of Prematurity	SC01	F	32	80
EB02	M	32	60	Birth	Retinitis Pigmentosa	SC02	M	29	50
EB03	M	47	70	Birth	Optic Nerve Hypoplasia	SC03	M	50	90
EB04	F	31	70	8 Months	Retinitis Pigmentosa	SC04	F	31	40
EB05	F	29	80	Birth	Microphthalmia	SC05	F	26	50
EB06	M	28	90	Birth	Congenital Toxoplasmosis	SC06	M	29	100
EB07	F	30	60	Birth	Agenesis	SC07	F	27	60
EB08	M	32	80	Birth	Leber Congenital Amaurosis	SC08	M	34	70
EB09	M	34	80	Birth	Anophthalmia	SC09	M	31	100
EB10	F	38	40	Birth	Retinopathy of Prematurity	SC10	F	41	60
EB11	F	28	70	Birth	Retinopathy of Prematurity	SC11	F	26	90
EB12	M	30	70	Birth	Leber Congenital Amaurosis	SC12	M	32	80
EB13	F	34	100	Birth	Retinopathy of Prematurity	SC13	F	31	90
EB14	F	32	100	2 Years	Retinoblastoma	SC14	F	31	70
EB15	F	35	60	4 Years	Retinitis Pigmentosa	SC15	F	37	100
EB16	F	32	60	Birth	Retinitis Pigmentosa	SC16	F	33	100

[a] Handedness was assessed with a modified version of the Edinburgh handedness questionnaire, which is also suitable for the blind population.
