# Supplementary material for: Similar object shape representation encoded in the inferolateral occipitotemporal cortex of sighted and early blind people
Source: PLoS Biol. 2023 Jul 25;21(7):e3001930. doi: 10.1371/journal.pbio.3001930 (PMC10368275; doi:10.1371/journal.pbio.3001930)
Supplement: S2 Table — (PDF) [file pbio.3001930.s012.pdf]

S2 Table. Stimuli

Words	English Translation
anello	ring
braccialetto	bracelet
candela	candle
ciotola	bowl
coperta	blanket
cucchiaio	spoon
cuscino	pillow
diario	notebook
fiammifero	matchstick
forchetta	fork
gessetto	chalk
lavagna	blackboard
moneta	coin
penna	pen
piatto	plate
portafoglio	wallet
portapenne	penholder
salvagente	lifebuoy
stuzzicadenti	toothpick
timone	rudder
tovagliolo	napkin
