# Supplementary material for: Similar object shape representation encoded in the inferolateral occipitotemporal cortex of sighted and early blind people
Source: PLoS Biol. 2023 Jul 25;21(7):e3001930. doi: 10.1371/journal.pbio.3001930 (PMC10368275; doi:10.1371/journal.pbio.3001930)
Supplement: S3 Table — (PDF) [file pbio.3001930.s013.pdf]

S3 Table. Inter-rater reliability of object properties and touch experience within each group of participants

Rating Items	EB (N = 16)	SC (N = 16)	IS (N = 16)
**Shape Similarity**	ICC = 0.953<br>CI = [0.943, 0.962]	ICC = 0.973<br>CI = [0.968, 0.978]	ICC = 0.973<br>CI = [0.968, 0.978]
**Conceptual Association**	ICC = 0.984<br>CI = [0.980, 0.987]	ICC = 0.985<br>CI = [0.982, 0.988]	ICC = 0.985<br>CI = [0.982, 0.988]
**Object Size**	ICC = 0.979<br>CI = [0.964, 0.990]	ICC = 0.994<br>CI = [0.989, 0.997]	ICC = 0.992<br>CI = [0.986, 0.996]
**Contextual Association**	ICC = 0.613<br>CI = [0.321, 0.817]	ICC = 0.856<br>CI = [0.747, 0.932]	ICC = 0.919<br>CI = [0.859, 0.962]
**Toolness**	ICC = 0.911<br>CI = [0.844, 0.958]	ICC = 0.893<br>CI = [0.812, 0.949]	ICC = 0.928<br>CI = [0.874, 0.966]
**Touch Experience**	ICC = 0.970<br>CI = [0.948, 0.986]	ICC = 0.975<br>CI = [0.956, 0.988]	ICC = 0.965<br>CI = [0.939, 0.984]

ICC: the intraclass correlation based on a mean-rating, consistency, two-way random model (i.e., ICC(C,k)) [1]

CI: 95% confidence interval of the true ICC

According to Koo & Li (2016) [2], Poor reliability: < 0.5; Moderate reliability: 0.5-0.75; Good reliability: 0.75-0.9; Excellent reliability: > 0.9

Reference

1. McGraw KO, Wong SP. Forming inferences about some intraclass correlation coefficients. Psychological Methods. 1996;1: 30–46. doi:10.1037/1082-989X.1.1.30

2. Koo TK, Li MY. A Guideline of Selecting and Reporting Intraclass Correlation Coefficients for Reliability Research. Journal of Chiropractic Medicine. 2016;15: 155–163. doi:10.1016/j.jcm.2016.02.012
