# Supplementary material for: Similar object shape representation encoded in the inferolateral occipitotemporal cortex of sighted and early blind people
Source: PLoS Biol. 2023 Jul 25;21(7):e3001930. doi: 10.1371/journal.pbio.3001930 (PMC10368275; doi:10.1371/journal.pbio.3001930)
Supplement: S4 Table — (PDF) [file pbio.3001930.s014.pdf]

S4 Table. Neural representation in bilateral aIPS

Three-way Mixed ANOVA *	Left aIPS		Right aIPS	
**Groups** (EB vs. SC)	F(1, 30) = 2.471	p = 0.126	F(1, 30) = 2.004	p = 0.167
**Tasks** (Shape vs. Conceptual)	F(1, 30) = 1.552	p = 0.223	F(1, 30) = 0.442	p = 0.551
**Representations** (Shape vs. Conceptual)	**F(1, 30) = 26.995**	**p < 0.001**	**F(1, 30) = 5.524**	**p = 0.026**
**Groups × Tasks**	F(1, 30) = 2.996	p = 0.094	F(1, 30) = 0.819	p = 0.373
**Groups × Representations**	F(1, 30) = 0.021	p = 0.885	F(1, 30) = 0.117	p = 0.735
**Tasks × Representations**	**F(1, 30) = 18.300**	**p < 0.001**	**F(1, 30) = 16.807**	**p < 0.001**
**Groups × Tasks × Representations**	F(1, 30) = 1.495	p = 0.231	F(1, 30) = 0.367	p = 0.549

* The Groups factor was between-subject, whereas Tasks and Representations were within-subject factors.
