# Supplementary material for: Similar object shape representation encoded in the inferolateral occipitotemporal cortex of sighted and early blind people
Source: PLoS Biol. 2023 Jul 25;21(7):e3001930. doi: 10.1371/journal.pbio.3001930 (PMC10368275; doi:10.1371/journal.pbio.3001930)
Supplement: S5 Table — (PDF) [file pbio.3001930.s015.pdf]

S5 Table. Neural representation in bilateral pIPS

**Three-way Mixed ANOVA ***	**Left pIPS**		**Right pIPS**	
**Groups**<br>(EB vs. SC)	$F(1, 30) = 2.925e\text{-}5$	$p = 0.996$	$F(1, 30) = 0.350$	$p = 0.559$
**Tasks**<br>(Shape vs. Conceptual)	$F(1, 30) = 0.840$	$p = 0.367$	$F(1, 30) = 0.517$	$p = 0.478$
**Representations**<br>(Shape vs. Conceptual)	**$F(1, 30) = 11.158$**	**$p = 0.002$**	**$F(1, 30) = 12.745$**	**$p = 0.001$**
**Groups × Tasks**	$F(1, 30) = 2.203$	$p = 0.148$	$F(1, 30) = 0.676$	$p = 0.417$
**Groups × Representations**	$F(1, 30) = 1.104$	$p = 0.302$	$F(1, 30) = 2.087$	$p = 0.159$
**Tasks × Representations**	**$F(1, 30) = 26.524$**	**$p < 0.001$**	**$F(1, 30) = 15.401$**	**$p < 0.001$**
**Groups × Tasks × Representations**	$F(1, 30) = 1.819$	$p = 0.188$	**$F(1, 30) = 4.597$**	**$p = 0.040$**

* The Groups factor was between-subject, whereas Tasks and Representations were within-subject factors.
