# Supplementary material for: Similar object shape representation encoded in the inferolateral occipitotemporal cortex of sighted and early blind people
Source: PLoS Biol. 2023 Jul 25;21(7):e3001930. doi: 10.1371/journal.pbio.3001930 (PMC10368275; doi:10.1371/journal.pbio.3001930)
Supplement: S6 Table — (PDF) [file pbio.3001930.s016.pdf]

S6 Table. Neural representation in bilateral vPMC

**Three-way Mixed ANOVA ***	**Left vPMC**		**Right vPMC**	
**Groups** (EB vs. SC)	F(1, 30) = 0.548	p = 0.465	F(1, 30) = 0.033	p = 0.858
**Tasks** (Shape vs. Conceptual)	F(1, 30) = 0.476	p = 0.496	F(1, 30) = 0.882	p = 0.355
**Representations** (Shape vs. Conceptual)	**F(1, 30) = 7.494**	**p = 0.010**	**F(1, 30) = 8.336**	**p = 0.007**
**Groups × Tasks**	F(1, 30) = 1.086	p = 0.306	F(1, 30) = 3.026	p = 0.092
**Groups × Representations**	F(1, 30) = 0.197	p = 0.660	F(1, 30) = 0.171	p = 0.682
**Tasks × Representations**	**F(1, 30) = 11.741**	**p = 0.002**	**F(1, 30) = 16.044**	**p < 0.001**
**Groups × Tasks × Representations**	F(1, 30) = 2.071	p = 0.160	F(1, 30) = 4.102	p = 0.052

* The Groups factor was between-subject, whereas Tasks and Representations were within-subject factors.
