# Supplementary material for: Similar object shape representation encoded in the inferolateral occipitotemporal cortex of sighted and early blind people
Source: PLoS Biol. 2023 Jul 25;21(7):e3001930. doi: 10.1371/journal.pbio.3001930 (PMC10368275; doi:10.1371/journal.pbio.3001930)
Supplement: S1 Text — (PDF) [file pbio.3001930.s017.pdf]

S1 Text. English translation of survey questions

Shape Familiarity
To what degree do you know the object's typical shape? (7-point Likert scale rating)
1: do not know it at all 7: know it very well

Conceptual Familiarity
To which degree do you know what this object is used for? (7-point Likert scale rating)
1: do not know it at all 7: know it very well

Touch Experience
How frequently have you touched this object? (7-point Likert scale rating)
1: have never touched it before 7: touch it every day

Size
How big is this object? (7-point Likert scale rating)
1: as small as a needle 7: as big as a television

Contextual Association
To which extent is this object associated with a specific context? (7-point Likert scale rating)
For example, a "cellphone" can occur in many different contexts and is unassociated with any particular cortex. You might rate 1 or 2. Instead, a "bowling ball" can only occur on the bowling alley; it is strongly associated with one specific context. You might rate 7.

Toolness
To which extent is this object a tool? (7-point Likert scale rating)
A tool is defined as a graspable and manipulable object that can transform the motor output into predictable mechanical actions for the purposes of attaining specific goals. You might rate 1 for "lamp," "chair," and "clock"; 7 for "hammer," "saw," and "drill."

Pairwise Shape Similarity
To which extent is this pair of objects similar in shape? (7-point Likert scale rating)
For example, "racket" and "pan" are similar in shape; you might rate 6 or 7; "racket" and "tennis ball" are not similar in shape, you might rate 1 or 2. Due to the recent expansion of business innovation, some objects may exist in a variety of shapes, e.g., watermelon may be in a square shape. Please rate the shape based on its most typical shape (i.e., spherical watermelon). Please disregard the other object properties, e.g., color, size, texture, and function.

Pairwise Conceptual Association
To which extent is this pair of objects conceptual associated? (7-point Likert scale rating)
For example, "racket" and "tennis ball" are conceptually associated; you might rate 7; "racket" and "pan" are not conceptually associated, you might rate 1. Please disregard the other object properties, e.g., color, size, texture, and shape.
