# Supplementary material for: Similar object shape representation encoded in the inferolateral occipitotemporal cortex of sighted and early blind people
Source: PLoS Biol. 2023 Jul 25;21(7):e3001930. doi: 10.1371/journal.pbio.3001930 (PMC10368275; doi:10.1371/journal.pbio.3001930)
Supplement: Attachment — Submitted filename: Response2Reviewer.pdf [file pbio.3001930.s020.pdf]

Modifications made to manuscript PBIOLOGY-D-22-02486 titled **Shared Neural Representations of Object Shape between the Sighted and Early Blind**.

We thank the editor and the reviewers for taking the time to read our manuscript thoroughly and for providing insightful comments that helped us significantly improve the quality of the study. In the following section, the comments are presented in black followed by our responses in blue.

Reviewer #1: The authors perform an fMRI study on blind and sighted participants to whom they present spoken words in the scanner that participants are asked to rate according to their shape similarity and contextual similarity. The authors find stronger responsiveness of the visual ILOTC region during the shape compared to the contextual task both in sighted and blind individuals. This study is interesting and advances the field, but needs revision with respect to the way the authors conceptualize their findings.

Thank you for the positive assessment and for giving us the chance to improve our paper based on your comments.

Major concerns

Q1. Concept. The concept that is here associated to the conducted analysis and obtained results is a bit misleading. The term "supramodal shape representation" suggests that shape representations are investigated that occur across modalities - however, the stimuli were only auditory. Given the authors start with saying that objects can be perceived by either vision or touch, and they do not mention in the abstract that in this study, stimuli were only auditory, the reader is led to believe that shape representation was tested in multiple modalities. This is similar to the first paragraph of the introduction, where the question is posed whether the brain can represent object information independent from the senses - this cannot be investigated testing on one sensory modality only. To test for supramodal representations of objects, one would imagine a task where specific objects are either haptically explored or heard, which would then assume to activate similar brain areas. Here, what the authors really investigate is to imagine the shape of an object or to imagine the context in which the object is used based on hearing the name of an object. Given that the shape task is a clear visual object feature task (elongated, angular, hollow, circular, discal) and the conceptual task is clearly related to object use (eating, writing, sleeping), it is not surprising that a visual area is activated more in the former condition. This, however, cannot be interpreted as a supramodal representation of objects. I would therefore suggest to adapt the concept and title to highlight that the ILOTC is involved in shape representation both in blind and sighted individuals. This also fits to the correlation to object size perception reported. How "supramodal" this representation is has to be clarified by other studies.

R1. We appreciate the reviewer's thoughtful comments, which concern the core motivation of this study. We clarify the rationale of this study as follows.

In the literature, it has been advocated that at least two criteria must be met to prove a traditionally considered visual cortex implements supramodal representation (please see the conditional definitions of

"supramodal" provided by Ricciardi et al., *Neuroscience & Biobehavioral Reviews*, 2014 in their Table on Page 73). First, as correctly pointed out by the reviewer, this region must be the overlapping area between visual and nonvisual tasks within the sighted population. Second, this region must be the overlapping area between the sighted and the early blind population in the nonvisual task.

The second criterion matters, as a purely visual brain area (e.g., V1) can also be activated in a nonvisual task due to visual imagery (e.g., Kosslyn & Thompson, 2003; Pearson, 2019). As the early blind has no visual experience, the representation of their "visual" cortex can be only derived from sensory modalities other than the visual one. Early blindness thus offers us a precious opportunity to rule out the factor of visual imagery to verify whether a traditionally considered visual area can truly represent information from other modalities as a supramodal brain region. This motivation lays the foundation for most studies testing early blind participants (e.g., Bedny & Saxe, 2012; Ricciardi et al., 2014; Bi et al., 2016; also see the fifth paragraph in the revised Introduction).

The current study chose words as stimuli. Since words, no matter visual or auditory, are arbitrary symbols bearing no obvious resemblance to the objects signified, they can be considered modality-neutral stimuli, potentially activating visual, haptic, or supramodal shape representation without inputting any modality-specific information and introducing any modality-specific bias (please see R2 for more details). Thus, as correctly pointed out by the reviewer, the current study did not use stimuli in different senses (i.e., visual and haptic objects) and, therefore, did not directly touch on the first criterion (i.e., the overlap of activity across the senses). Nevertheless, it is built on already well-documented evidence about the phenomenon of the lateral occipital tactile-visual complex (LOtv). This region, located in the inferolateral occipitotemporal cortex (ILOTC), shows stronger activation when participants both see or touch objects in comparison to shapeless textures (e.g., Amedi et al., 2001, 2002; Stilla & Sathian, 2008; Snow et al., 2015). Given that the ILOTC has already been proven to meet the first criterion, the current study aims to test whether it also meets the second one. Only when the ILOTC represent shape in the early blind can the conclusion of supramodal representation be reached.

The reviewer pointed out that *what the authors really investigate is to imagine the shape of an object or to imagine the context in which the object is used based on hearing the name of an object. Given that the shape task is a clear visual object feature task (elongated, angular, hollow, circular, discal) and the conceptual task is clearly related to object use (eating, writing, sleeping), it is not surprising that a visual area is activated more in the former condition.* This might be self-evident for the sighted population but not necessarily true for the early blind one. If the ILOTC is not a supramodal region but a purely visual one, the ILOTC in the early blind will not represent object shapes due to a lack of visual experience. Then, this region in the early blind will not be activated more in the shape task and will not represent shape similarity in the RSA analysis. Our results falsified the purely visual hypothesis, leading to a supramodal conclusion.

The only flaw of the above logical chain is that instead of a supramodal representation in the ILOTC of the sighted population, it might be possible that the ILOTC in the sighted population represents visual shapes, whereas the ILOTC in the blind population represents haptic shapes. Our connectivity analysis can partially clarify this issue. We found that the ILOTC in both sighted and blind had strong connectivity to the haptic network in the frontoparietal regions. It is thus unlikely that the sighted population only represents the shape visually.

Converging the evidence in the previous literature and the contribution of the current study, we believe that suggesting the ILOTC is a supramodal region is the most parsimonious conclusion we can arrive at. However, we also understand the reviewer's concern that our study alone cannot lead to this conclusion. We thus changed our title to "Shared Neural Representations of Object Shape between the Sighted and Early Blind," which better fits the content of the study. In the current Introduction, we motivated our study to test the other three alternative hypotheses other than the supramodal hypothesis. In the revised Discussion, we emphasized why the supramodal hypothesis is the most parsimonious explanation based on the evidence in the current study and previous literature.

Q2. In a similar vein, the authors pose the hypothesis in the introduction that potentially, the ILOTC may be more responsible for conceptual representations in blind compared to sighted individuals. This is still possible as one could imagine that the representation of the object's shape induced by haptic object exploration is more pronounced in ILOTC in blind compared to sighted individuals. This could only be tested by an experiment that presents the same object in different modalities, as pointed out above. This hypothesis can therefore also not be clarified in this study, which requires re-writing the introduction in parts.

R2. We apologize if our reasoning was not clear in the previous version of the manuscript. We proposed that the ILOTC may be responsible for the conceptual representation in blind individuals, not because *the representation of the object's shape induced by haptic object exploration is more pronounced in ILOTC in blind compared to sighted individuals.* We find no evidence in the literature to support it. Whether the ILOTC in the early blind can have a more pronounced shape representation through haptic exploration is also beyond the scope of this study.

The current study focuses on whether the ILOTC implements shape or conceptual representation in the sighted and early blind. We chose words instead of haptic objects as our stimuli to meet this purpose. To clarify this point, we have revised the relevant paragraph in our introduction (Page 6, Line 125-141): *To address these unsolved questions comprehensively in a single study, we used functional Magnetic Resonance Imaging (fMRI) to characterize the brain activity of sighted and early blind participants when they were performing both shape and conceptual verification tasks on the same set of auditory words referring to manmade objects. Univariate contrast between shape and conceptual tasks was performed to localize brain areas specific for shape or conceptual processing. We chose words instead of haptic objects as stimuli because words are arbitrary symbols bearing no obvious resemblance to the objects signified. That means the words, per se, do not carry object information and can elicit shape and conceptual representations without bias. In contrast, haptic objects carry shape information. The participants would have to process the shape information to recognize the objects in both shape and conceptual tasks, and we would no longer isolate the shape representation by contrasting the shape task with the conceptual task.*

We suspected that the ILOTC might be responsible for the conceptual representation because it has indeed been hypothesized that the "visual" cortex of the early blind could "switch function" and be involved in functions that are not linked to what the region does in sighted individuals, like lexical semantics (see Bedny, 2017, for review). We have rephrased the paragraph in the revised manuscript to make our reasoning more explicit (Page 6, Line 113-123): *The pluripotent neuroplasticity hypothesis predicts that the "visual" cortex in the early blind, due to a lack of visual input since birth, could repurpose its function*

for cognitive faculties that are distant from its native computation in vision, like language or mathematics (see Bedny, 2017 for review). This neurofunctional reorganization process usually accompanies enhanced connectivity between the "visual" cortex in the early blind and high-order brain systems (Bedny, 2017). In line with this hypothesis, it has been reported that the "visual" cortex in the early blind is more sensitive to lexical semantics than the sighted participants (e.g., Noppeney et al., 2003; Bedny et al., 2011). Moreover, the activity in the lateral occipital cortex in the early blind is more synchronized to the areas in the perisylvian language network than in the sighted participants (Bedny et al., 2011). It is thus possible that the lLOTC in the early blind implements conceptual instead of shape representation due to functional reorganization.

Q3. It is also not clear to me why the authors refer to the conceptual task as "linguistic". When hearing the word of an object, and being asked whether this object is used for eating, sleeping, writing etc, this is not a semantic task but it is really about imaging the use of the object in everyday life. The interpretation of this being a "semantic" task is therefore highly speculative. This can be mentioned in a balanced way in the discussion, but should for sure not be reported as a result in the results section (line 278 and subsequent, see also line 319 where the authors also give a strong and rather questionable interpretation of the conceptual task being "higher-level cognitive"). This needs to be changed in the results and discussion sections.

R3. We appreciate that the reviewer brings up this crucial issue. We fully agree that conceptual representations are not only supported by language coding but also by the experience-based one, which is suggested by most of the semantic theories (e.g., Paivio, 1986; Barsalou et al., 2008; Mahon & Caramazza, 2008; Dove, 2009, 2011; Vigliocco et al., 2009; Zwaan, 2014; Reilly et al., 2016; Xu et al., 2017; Bi, 2021).

We also agree with the reviewer that a balanced discussion acknowledging both codings of conceptual association is essential. In the discussion, we now allocated an independent paragraph talking about experience-based coding, which was missing in the previous version (Page 26, Line 619-630): *The exception for the AG indicates that conceptual associations can be represented in a format other than linguistic. Previous studies have shown that the AG is not a purely linguistic region but also part of the default mode network engaged in memory-based simulation (e.g., Buckner et al., 2008; Xu et al., 2016, 2017). Compared to the other linguistic regions, the AG is less responsive to word forms (e.g., Graves et al., 2022) but more sensitive to the retrieval of multimodal episodic memories (e.g., see reviews by Humphrey et al., 2021). It is thus possible that the AG codes thematic relations based on the spatiotemporal continuity in our sensorimotor experience (e.g., hammers and nails often co-occur; Mirman et al., 2017), which is apt to reflect on activity patterns (Xu et al., 2018), in contrast to the coding based on linguistic associations in the other language areas. In line with this idea, we found other brain regions in the default mode network also representing conceptual associations in the whole-brain searchlight RSA analysis, including the left precuneus and the left dorsal medial prefrontal cortex (Supplementary Figure 8).*

We have also carefully gone through the manuscript to ensure that we have not referred to the conceptual task as "linguistic" or “higher-order”, as rightly pointed by the reviewer, these attributions were speculative and ambiguous. What we instead did articulate in the Results section is that our univariate analysis shows that the conceptual task compared to the shape task, activated a brain network that neatly

matched the left-lateralized perisylvian language network (Fedorenko et al., 2010, 2011; Friederici, 2011), including the anterior portion of the temporal lobe, the superior temporal gyrus, the supramarginal gyrus, the angular gyrus, and the ventral part of the inferior frontal gyrus. This result suggests that language-based coding plays a crucial role in our conceptual task (see discussion and also Xu et al., 2017; Bi et al., 2021). To provide the readers an intuitive view of the overlap between the language network and the brain activation in our conceptual task, we added another figure in our revised manuscript (Page 16, Line 357-361): *These regions neatly matched the language network (Fedorenko et al., 2010, 2011) with the absence of the triangular part of the IFG and the 55b region in the premotor cortex (Glasser et al., 2016) (see the overlap in Supplementary Figure 5), in line with previous studies suggesting these two dorsal regions play a non-semantic role in language processing (e.g., Hickok & Poeppel, 2007; Binder et al., 2009; Hickok et al., 2022).*

As the early "visual" cortex in the early blind was also more activated in the conceptual task than in the shape task, we suspected that the "visual" cortex in the early blind might repurpose itself to a similar role as the language network played, in line with abundant evidence that the "visual" cortex in the early blind is involved in semantic and grammar processing (e.g., Bedny et al., 2011; Lane et al., 2015; Van Ackeren et al., 2018). In the revised manuscript, we have deleted the vague word "higher-level" and made our meanings more explicit (Page 15, Line 330-332): *This finding suggests that the earlier "visual" cortex in EB (i.e., the left cuneus) might repurpose itself to a similar role as what the language network played in the conceptual task (see also a recent meta-analysis by Zhang et al., 2019).*

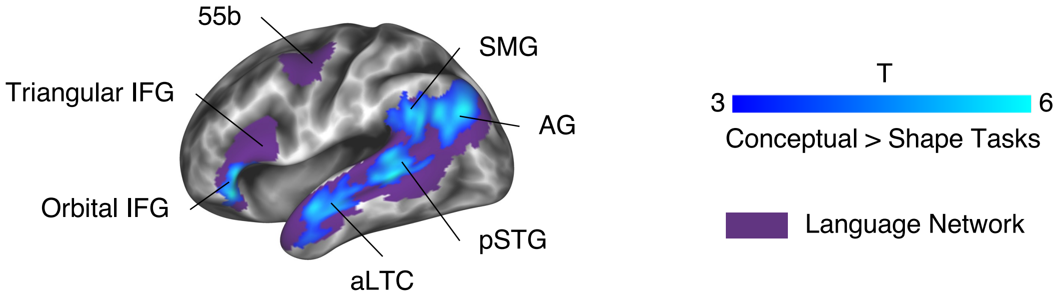

Supplementary Figure 5. The conceptual task in our study involved a brain network (in blue) almost identical to the language network (in purple), except for the triangular part of the IFG and the 55b area in the premotor cortex. These two dorsal regions are considered to play a non-semantic role in language processing. The conceptual network (in blue) was defined in the contrast between the conceptual task and the shape task with the control of other object properties (N = 48; vertex-wise $p < 0.001$, cluster-level FWE corrected $p < 0.05$). The language network (in purple) was defined in the study by Fedorenko et al. (2010) with the data updated from 220 participants. The overlap coefficient between these two networks was 83.05%. Such highly overlapped results suggest that the language system plays a crucial role in our conceptual task.

Q4. Results. The interaction between group and task was reported as non-significant with a p-value of 0.123. Given the groups were relatively small, there may be an interaction effect that is not fully revealed here based on undersampling. It would therefore be good if the authors would report the group size that would be needed to find a significant interaction effect, and if they would perform a Bayesian analyses showing that indeed the absence of an effect has a higher likelihood that the presence of an effect. In addition, it should be reported in the text where this trend towards an interaction comes from.

R4. We understand the reviewer's concern. In the analysis mentioned by the reviewer, we investigated the interaction between Groups (EB vs. SC) and Tasks (shape vs. conceptual tasks) through a linear mixed model, in which participants were the grouping factor of the reaction time (RT) of multiple trials for each task. We would have liked to use the Bayesian method following the reviewer's advice. However, the Bayesian linear mixed model method is still under development, and how to estimate the Bayesian factor of linear mixed model is under debate (e.g., van Doorn et al., 2021). Currently, neither the *brms* package in *R* nor *JASP* provides the Bayesian factors for the Bayesian linear mixed model.

We are also aware that a post hoc power analysis might not be the best solution to reveal the underpower problem. It will be a circular reference (see this blog: https://tinyurl.com/ytxtz7jx). Insignificant effects will always be *undersampling,* and with a sample size *big enough,* all effects could be significant unless the effect on the entire population is strictly nonexistent. A better way is to provide the effect size instead of the observed power. Although effects with small effect sizes might also be interesting, there is no way of knowing whether such inter-subject variability is due to actual variations in an effect or measurement of noise.

The software we used to calculate the statistics in the manuscript (i.e., *JASP*) does not provide the effect size for the mixed linear model. To estimate the effect size of the interaction effect, we re-ran the linear mixed model using the *lme4* package in *R* and calculated the effect size as partial η^2 using the *effectsize* package in *R*. The F and p values of Groups (EB vs. SC), Tasks (shape vs. conceptual tasks), and the interaction between Groups and Tasks were identical to those calculated using *JASP* reported in the manuscript. The effect size (i.e., partial η^2) of Groups was 0.02, Tasks was 0.71, and Interaction was 0.08.

According to Miles & Shevlin (2001), small effect: $\eta^2 > 0.01$; medium effect: $\eta^2 > 0.06$; and large effect $\eta^2 > 0.1$;. the interaction effect between Groups and Tasks, therefore, was a medium effect. So we agree with the reviewer that it might be worth reporting such a marginally insignificant result, and we have revised the manuscript accordingly (Page 12, Line 257-264): *The analysis revealed a significant task effect ($F(1,30) = 73.055$; $p < 0.001$), whereas the group effect was found insignificant ($F(1,30) = 0.732$, $p = 0.399$), and the interaction effect between groups and tasks had only a slight trend toward significance ($F(1,30) = 2.552$, $p = 0.123$). The significant difference between shape and conceptual tasks aligns with the evidence suggesting that retrieving specific semantic features (e.g., shape knowledge) requires more time than general semantic knowledge (e.g., function knowledge; Hauk, 2016). The interaction effect showed a weak trend that the shape task was slightly more difficult than the conceptual task for the EB than the SC ([(EB > SC) × (shape > conceptual tasks)]; $z = 1.588$, $p = 0.112$), which might be due to a lack of visual experience.*

Q5. The same is true for the comparison between SC-SC and EB-SC, where a p-value of 0.073 is interpreted as "no effect". Please apply the same methods as suggested above here, and adapt the interpretation based on the results of these analyses.

R5. The analysis mentioned by the reviewer was a two-way ANOVA between Tasks (shape vs. conceptual tasks) and Group Pairs (EB-EB vs. SC-SC vs. EB-SC) in the left ILOTC. We were able to implement a Bayesian analysis for the ANOVA using JASP 0.17.1. Table 1 shows moderate evidence for the effect of group pairs ($BF_{10} = 3.842$), consistent with the traditional statistical testing. Table 2 further shows the

results of post hoc comparisons between the group pairs. We had anecdotal evidence of the difference between EB-EB and SC-SC ($BF_{10} = 1.908$), suggesting the representational pattern within the SC group was more coherent than those within the EB group. Evidence for the differences between EB-EB and EB-SC and between SC-SC and EB-SC shows the tendency to favor the null hypothesis ($BF_{10} < 1$). Moderate evidence shows no differences between EB-EB and EB-SC ($BF_{10} = 0.283$), and anecdotal evidence shows no differences between SC-SC and EB-SC (BF10 = 0.769).

Since a BF10 of 0.769 only showed anecdotal evidence in favor of the null hypothesis when testing differences between SC-SC and EB-SC, we have not concluded in the revised manuscript that there was significant evidence of the null results. It is however safe to suggest that the results demonstrate *no significant evidence showing a boundary effect between the neural representations across groups* (Page 19, Line 432-433).

Table 1: Two-way ANOVA in the left ILOTC.

Effects	P(incl)	P(excl)	P(incl|data)	P(excl|data)	BF_{incl}
Tasks	0.400	0.400	0.758	6.709×10^{-13}	$1.130\times10^{+12}$
Group Pairs	0.400	0.400	0.602	0.157	3.842
Tasks ✻ Group Pairs	0.200	0.200	0.242	0.602	0.402

Note. Compares models that contain the effect to equivalent models stripped of the effect. Higher-order interactions are excluded. Analysis suggested by Sebastiaan Mathôt.

Table 2: Post Hoc Comparisons – Group Pairs

		Prior Odds	Posterior Odds	$BF_{10,\,U}$	error %
EB-EB	EB-SC	0.587	0.166	0.283	0.012
	SC-SC	0.587	1.121	1.908	0.009
EB-SC	SC-SC	0.587	0.452	0.769	0.010

Note. The posterior odds have been corrected for multiple testing by fixing to 0.5 the prior probability that the null hypothesis holds across all comparisons (Westfall, Johnson, & Utts, 1997). Individual comparisons are based on the default t-test with a Cauchy (0, r = 1/sqrt(2)) prior. The "U" in the Bayes factor denotes that it is uncorrected.

P.S. We evaluated the Bayesian Factor based on the cutoff from Andraszewicz et al., 2015.

Bayes Factor	Interpretation
> 100	Extreme evidence for alternative hypothesis
30 – 100	Very strong evidence for alternative hypothesis
10 – 30	Strong evidence for alternative hypothesis
3 – 10	Moderate evidence for alternative hypothesis
1 – 3	Anecdotal evidence for alternative hypothesis
1	No evidence
1/3 – 1	Anecdotal evidence for null hypothesis
1/3 – 1/10	Moderate evidence for null hypothesis
1/10 – 1/30	Strong evidence for null hypothesis

1/30 – 1/100	Very strong evidence for null hypothesis
< 1/100	Extreme evidence for null hypothesis

Q6. Discussion. Based on the comments and suggestions mentioned above, the discussion needs to be adjusted.

R6. We have revised the discussion thoroughly based on the reviewer's comments. Please see our previous responses.

Q7. Minor comments

In Figure captions, the short forms EB and SC are not explained

R7. Thanks for pointing it out. We have now explained them in the figure captions.

Reviewer #2: In this work, Xu and colleagues investigate whether the modality-independent recruitment of the inferior lateral occipitotemporal cortex (ILOTC) relates to the processing of shape features rather than to the conceptual representation of objects. To address this question, the authors ask early blind and sighted individuals to perform a shape categorization task (e.g., round vs. square shape) and a conceptual verification task (e.g., it is for eating vs. writing) on 21 manmade objects while their brain activity is recorded using fMRI. Shape and conceptual similarity judgments collected in behavioral experiments complement the imaging experiment, and brain data are analyzed using univariate and multivariate (i.e., RSA) methods.

Results demonstrate (1) that ILOTC is recruited to a greater extent when participants focus on shape features, (2) that the response pattern in this region relates to the similarity in shape more than to the similarity in conceptual features, and (3) that this area is specifically connected at rest with a network of shape-relevant frontal and parietal areas. Importantly, these results are observed in sighted and early blind individuals.

Overall, this is a very well-conducted study and a relevant contribution to the literature on the brain representation of object features. The study hypothesis is clearly explained, the experimental paradigm is well-conceived, and the analyses are sound. The results are interesting, and their interpretation is convincing.

Thank you for recognizing the value of our work.

Here, I am providing a list of comments that the authors may want to incorporate in a revised version of the manuscript.

Q1. I believe the paragraph about parietal regions (lines 56-69) in the "Introduction" section could be moved to the discussion section. Because the focus of this investigation is ILOTC (e.g., LOtv peak coordinates extracted from previous studies are used as a reference), this paragraph sounds like an unnecessary digression from the main topic.

R1. We share the same feeling as the reviewer. The transition was not coherent enough, and the paragraph about haptic shape representation seemed off the road. However, we do need a paragraph offering the readers some necessary contexts about the haptic shape literature since the frontoparietal regions showing higher univariate activation levels in the shape task than in the conceptual task were expected based on former literature (e.g., Binkofski et al., 2001; Dettmers et al., 2003; Hömke et al., 2009), and it also allows us to introduce the motivation for the resting-state functional connectivity analysis showing the connection between the ILOTC and the regions reported to implement haptic shape representation.

To increase the cohesion of this paragraph with the rest of the introduction, we have revised the introduction as follows (Page 4: Line 71-77): *In addition to the frontoparietal circuit, haptic shape perception intriguingly involved the anterolateral part of the LOC, a region located in the inferolateral occipitotemporal cortex (ILOTC); this region shows stronger activation when participants both see or touch objects in comparison to shapeless textures (e.g., Amedi et al., 2001, 2002; Stilla & Sathian, 2008; Snow et al., 2015). Based on this unique multisensory property, researchers termed the ILOTC region the*

lateral occipital tactile-visual complex (LOtv; Amedi et al., 2002) and suggested it implements supramodal shape representation (Ricciardi et al., 2014).

Q2. The paragraph detailing the fMRI paradigm in the "Materials and Methods" section is not sufficiently clear to me (lines 765-789). I feel adding a figure that recapitulates the fMRI paradigm would help in clarifying the structure (e.g., randomization of task conditions) and timing of the different experimental phases (e.g., probe, stimulus presentation, participant response).

R2. Thank you for the suggestion. We have now added a figure illustrating the fMRI procedure (Supplementary Figure 1, also below).

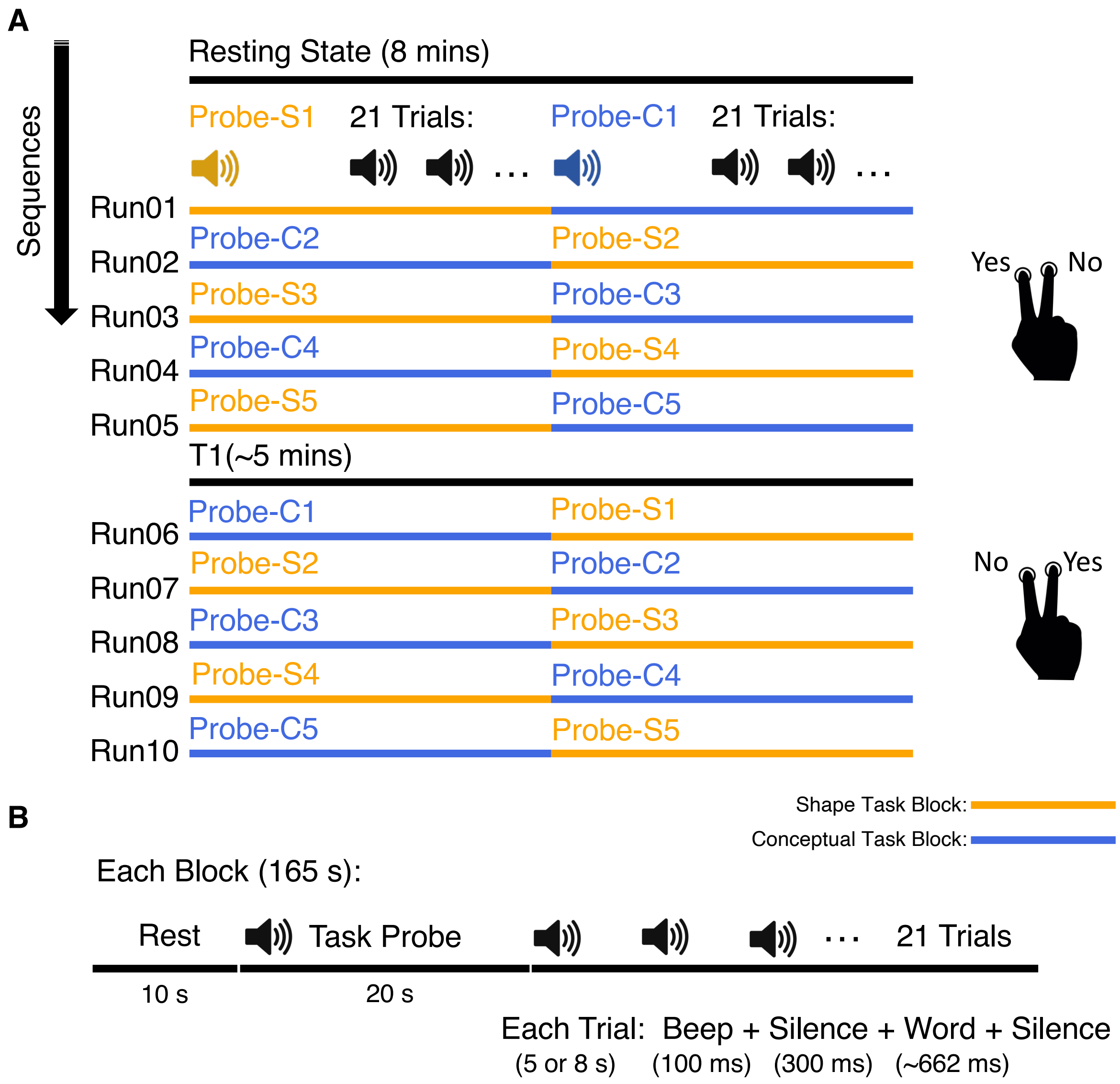

Supplementary Figure 1. The procedure of the fMRI experiment. (A) The structure of the fMRI scanning session. The order of the two task blocks was interleaved across runs within each subject. Whether the first run started with a shape or a conceptual block was counterbalanced across subjects within the early blind and the sighted control group. Task probes S1 to S5 randomly corresponded to five questions about objects' shape for each participant (i.e., is the object elongated, angular, hollow, circular, and disc-shaped?). Task probes C1 to C5 randomly corresponded to five questions about objects' function for each participant (i.e., is the object used for eating, writing, sleeping, lighting, and purchasing?). The button configuration (correspondence between yes/no judgments and index/middle fingers) in the first five runs was counterbalanced across subjects within the early blind and the sighted control group. The button configuration was switched in the second set of five runs (after T1 acquisition) for each participant. (B) The timing of each block and each trial. The participants were instructed to respond by pressing buttons within 5 s.

Q3. Concerning the assessment of statistical significance, the authors write: "We performed the group-level one-sample t-test or two-sample t-test[...]. Five thousand sign-flips were performed[...]" (lines 958-964). To my knowledge, sign-flipping is used only in the case of one-sample or two-sample paired non-parametric t-test (after having computed the difference between time points for each participant). Thus, it is not clear what Xu and colleagues mean when they refer to sign flips in the context of a two-sample unpaired t-test. Do they mean the shuffling of labels?

R3. We appreciate very much that the reviewer raised this question. It is true that, intuitively, one-sample tests and paired tests are prone to use the sign-flipping method assuming the independent and symmetric errors (ISE), while two-sample tests should use the permutation method assuming the exchangeable errors (EE) (i.e., shuffling labels). However, two-sample tests can also use the sign-flipping method. As we were contrasting the two groups (contrast matrix: 1 -1), flipping signs of a random subsets of individuals can also produce a valid null distribution.

While both methods suit two-sample tests, the permutation method assuming EE requires that the variances of the two groups are equal, whereas the sign-flipping method assuming ISE does not have this requirement. The following are two relevant sentences cited from Winkler et al. (2014), the theoretic paper on neuroimaging permutation inference and the reference of the PALM software:

Page 383: *Independent and symmetric errors (ISE) can be considered for measurements that arise, for instance, from differences between two groups if the variances are not assumed to be the same.*

Page 384: *The choice between EE and ISE depends on the knowledge of, or assumptions about, the error terms. Although the EE does not require symmetry for the distribution of the error terms, it requires that the variances and covariances of the error terms are all equal, or have a structure that is compatible with the definition of exchangeability blocks (discussed below).*

To ensure we understood correctly, we contracted Dr. Winkler (the first author of Winkler et al., 2014), and he confirmed our understanding. Here is our communication on Github: https://github.com/andersonwinkler/PALM/issues/42#issue-1571133032.

We chose the sign-flipping method instead of the permutation one because the variance of the early blind, on many occasions, is greater than the variance of the sighted control. There are studies explicitly targeting this issue (Rosenke et al., 2020; Sen et al., 2022), and this phenomenon also occurred in our study (Figure 4C: the coherence of representational pattern in the sighted control was more significant than the coherence in the early blind in the left ILOTC).

To avoid any possible misunderstanding, we added the following sentences to the method (Page 43: Line 1028-1032): *It is worth noting that, in the two-sample tests, we also chose the sign-flipping method assuming independent and symmetric errors instead of the traditional permutation method assuming exchangeable errors. This is because the variance of the early-blind group, on many occasions, is greater than the variance of the sighted-control group (Figure 4C; e.g., Rosenke et al., 2020; Sen et al., 2022), which violates the equal variance assumption of exchangeability.*

Q4. Although the results on the modality- and experience-independent representation of shape in ILOTC are convincing and in line with previous findings, the fact that the authors have used spoken words to trigger mental representations of objects limits the possibility of understanding which shape features are actually encoded in ILOTC. For instance, even though silhouette, curvature, and medial axis often correlate with each other, they may contribute differently to the final percept and could be mapped in distinct brain areas (see Papale et al., 2020; DOI: 10.1152/jn.00212.2020). In particular, medial-axis - a property that well accounts for behavioral similarity judgments and transformation-resistant shape descriptors - seems to be encoded in LO. I believe the discussion section would benefit from a more thorough analysis of the potential impact that having used memory-related activations of mental representations of objects, rather than actual perception (e.g., tactile match to sample vs. tactile recognition tasks), may have had on the present findings.

R4: We thank the reviewer for this valuable comment, which inspires us to think more deeply about the differences in shape representation between the LO and the ILOTC in light of the new results coming from this study. We now have complemented this part in our discussion:

In Page 23, Line 546-561, we argued that the ILOTC might represent schematic or prototypic shape of an object concept derived from various concrete shape exemplars represented in the LO of the sighted people: *On the one hand, the ILOTC (mainly in BA 37) is anterior to the lateral occipital cortex (LO, mainly in BA 18), a visual shape perception region representing shape features like curvatures and medial axes, in contrast to the earlier visual cortex implementing retinotopic representation (e.g., silhouettes) (e.g., Vernon et al., 2016; Papale et al., 2020). According to embodied semantic theories (Barsalou et al., 2003) and the "anterior shift" phenomenon noted first by Thompson-Schill (2003), the associative cortex anterior to each sensorimotor area can gradually capture the regularities of the activity patterns in its nearby sensorimotor cortices induced by different exemplars of the same concept (e.g., different exemplars of an apple) and generate a schema-like representation as the sensorimotor knowledge of that concept (e.g., the typical color, shape, and action related to an apple). In line with this hypothesis, previous studies have shown that the region representing objects' color knowledge is localized to the fusiform gyrus anterior to the color perception area in V4 (Miceli et al., 2001; Simmons et al., 2007), and language-induced category-specific activations are aligned with but anterior to the visual-induced activations of the same semantic category (Popham et al., 2021). The ILOTC, which is anterior to the LO, thus possibly*

represents objects' shape knowledge—the schematic or prototypic shape of an object concept —derived from various concrete shape exemplars represented in the LO of the sighted people.

In page 25, Line 575-581: We further considered what a supramodal representation would be like in light of recent results: *Given this supramodal nature, the ILOTC might not only schematize the visual shape representation from the LO but also integrate the haptic shape representation from the IPS-vPMC circuit by amplifying the "affordance" shape features utilized for object grasping. This hypothesis is supported by the evidence that the ILOTC is more sensitive to pictures of graspable tools over non-graspable manmade objects (e.g., Bracci et al., 2013) and its activity pattern better reflects the shape of objects' handles than their functional parts (Wu et al., 2020).*

Reviewer #3: The report by Xu et al., "Supramodal Shape Representation in the Human Brain," presents results from a study of the representation of manipulable and graspable manmade objects in the brains of early blind and sighted control participants that investigates whether the supramodal representation in the the lateral occipital tactile visual complex (LOtv), and more broadly the inferior lateral occipital temporal complex (ILOTC) reflects information about shape, conceptual knowledge, or both. The study analyzed neural responses, as measured with fMRI, to auditory words for 21 objects, during tasks that focused processing on shape or conceptual attributes. Results from both univariate and RSA analysis show strong evidence for shape representation that is independent of conceptual features, and the authors conclude that ILOTC represented shape similarity, not conceptual association, in both the early blind and sighted control participants.

Q1. This is a well-designed and comprehensive study, and the conclusion that the representation in ILOTC reflects supramodal shape information is well-supported. The other conclusion, however, that "reject[s] the alternative hypotheses that such activation depends on visual imagery or conceptual processing," is too strong. "Conceptual processing" here is operationalized in a very narrow domain, namely concepts concerning the typical settings in which manipulable and graspable manmade objects are encountered. It is well-known that conceptual information is encoded in LOC cortex. The domains of the conceptual knowledge that have been clearly demonstrated, however, concern entities that are not relevant to tactile-visual representation, namely animal taxonomy, animacy, agency, and behavior (Kiani et al. 2007; Connolly et al. 2012; Sha et al. 2015; Nastase et al. 2017; Thorat et al. 2019). These entities and concepts are typically not experienced through touch and their representation in the early blind may be, as of now, largely unexplored. The full range of conceptual knowledge about manmade objects also is not well-sampled by their task, which concerns only the contexts in which these objects are encountered, but not how they are manipulated, what is their function, what are they made of, what is their typical color, etc. The neural representational geometry of conceptual associations among manmade objects is not well understood. In Kiani et al.'s seminal study in monkeys, they found a highly meaningful conceptually-driven geometry in monkey IT cortex for animals but nothing for objects, which could be attributed to monkey's limited knowledge about manmade objects. The results in the current study show that the representational geometry of conceptual associations is mostly negative except, curiously, for areas in the default mode system (TPJ, precuneus, MPFC) that were also involved in representation of shape knowledge.

Consequently, the conclusion of this study cannot address the representation of conceptual information in LOC more broadly. Their conclusion supporting a negative hypothesis, therefore, should be more carefully focused on the limited conceptual domain that they studied and not generalized to representation of conceptual knowledge in LOC.

R1. We thank the reviewer for recognizing the value of our work. We fully agree with the reviewer's thoughtful comment that this study only focused on one specific type of conceptual knowledge (i.e., thematic knowledge or conceptual associations). As pointed out by the reviewer, our conclusion cannot extend to other types of knowledge, like taxonomic knowledge. We have made the following revisions to address this point in the manuscript.

First, in the discussion, we have added one paragraph to emphasize the scope of the current study and explain why it is challenging to identify taxonomic representations at the conceptual level (Page 27, Line 632-649): *It is worth noting that this study only focused on one type of conceptual knowledge: thematic*

relations or conceptual associations based on functional knowledge ("plate" and "fork" used for dining vs. "pillow" used for sleeping). The conclusion, therefore, cannot extend to other knowledge types like taxonomic categories ("plate" and "pillow" as manmade objects vs. "dog" as animals). In the "Stimuli" section, we elaborated on why we narrowed down the stimuli to one taxonomic category (i.e., the manmade objects) and only focused on thematic relations. One of the main reasons is that it is challenging for a neuroimaging study to distinguish whether a brain area represents taxonomic knowledge per se or simply shows a preference for the features of specific categories. Take the ILOTC as an example. Previous studies have shown that regions overlapping or superior to the ILOTC are more sensitive to manmade objects than the other categories even in early blind participants (e.g., Peelen et al., 2013; Wang et al., 2015; Dormal et al., 2018; Mattioni et al., 2020). However, this does not necessarily mean the ILOTC represents taxonomic knowledge at the conceptual level. Instead, our results suggest that the ILOTC represents shape knowledge derived from both visual and haptic modalities, and its potential preference for manmade objects is likely perceptual. Either because people have more haptic experiences with manmade objects or the manmade objects have more affordance shape features for grasping, the ILOTC can receive additional shape information from haptic modalities and therefore become more sensitive to the shapes of manmade objects (see previous discussions).

Second, in the section introducing stimuli selection, we have rewritten the paragraph to further clarify why we narrowed down the stimuli to one taxonomic category (i.e., the manmade objects) and only focused on thematic relations (Page 31, Line 724-753): *This study focused on thematic relations by confining its stimuli to one taxonomic category—manmade objects, based on the following considerations: (1) Growing evidence suggests taxonomic and thematic relations rely on dissociable mental and neural systems (e.g., Mirman et al., 2017; Xu et al., 2018). Confusing two quality-different conceptual relations into one unified conceptual RDM might be problematic. (2) It is challenging for a neuroimaging study to distinguish whether a brain area represents taxonomic knowledge per se or shows preferences for specific taxonomic categories. Such distinction matters. The former assumes a dedicated brain area representing taxonomic relations among concepts, whereas the latter could mean a brain area representing category-specific features at the pre-conceptual stage. The category-specific features could be the distinguishing features across brain systems. For example, as mainly manmade objects have manipulation-related features and humans have social features, brain systems processing action or social information will exhibit taxonomic preference. The category-specific features could also be within one brain system. For example, different patches along the visual pathway show category-specific effects (e.g., Kiani et al., 2007; Kanwisher, 2010) serving the perceptual purpose (e.g., Grill-Spector & Weiner, 2014). However, in both cases, we can hardly say these brain systems represent taxonomic relations among concepts. Taxonomic representation at the conceptual level is assumed to emerge from the converge zones when multiple features have already been bound onto a concept (e.g., Rogers et al., 2004; Mirman, 2017). (3) Some hypothesis argues that category-specific representations at the conceptual level do exist; however, they cannot be represented in local brain areas but emerge from the connectivity among distributed categorical-specific regions across different brain systems (Mahon & Caramazza, 2011). This hypothesis explains why category-specific semantic deficits are well-documented in neuropsychological literature (e.g., Capitani et al., 2003; Caramazza & Mahon, 2003) but are difficult to localize in the brain. However, category-specific representation at the conceptual level does not equal taxonomic knowledge representation, and the proposed connectivity-based neural representations are beyond the scope of this study. (4) Compared to the sighted, the early blind lack perceptual experience with many concepts in the natural world and have different neural representations of these "imperceptible" concepts (Striem-Amit et al., 2018). Using only*

manmade objects ensures a relatively fair comparison between the sighted and the early blind (see ratings on touch experience on Figure 1D).

Third, in the sentence the reviewer mentioned, we have specified the type of conceptual knowledge we focused on (Page 22, Line 517-519): *Our results thus favor the hypothesis suggesting the ILOTC implements supramodal shape representation and argue against the alternative hypotheses that such activation depends on visual imagery or* *conceptual associations based on functional relevance.*

Minor points.

Q2. Line . The role for the 16 independent sighted (IS) participants is unclear. They are included in results for all 48 participants, but results restricted to this group are not presented.

R2. We originally planned to use these 16 sighted participants to match our potential early blind participants whom we knew of, and some of them already took part in our past experiments. However, further recruitment of blind participants was stalled due to Covid-19 restrictions, and we could not find age-, gender-, and handedness-matched early blind participants for these sighted participants. We pooled all 48 subjects when there were no significant differences between the 16 early blind participants and their 16 matched sighted controls because we wanted to maximize the power and present the readers with the most reliable results by utilizing all the data. We did not provide the results restricted to the IS group, as their results are consistent with those from all the subjects but less significant. Here, we will show the results of the most important findings in this study restricted to IS group.

Figure 1 shows the results from the univariate contrast between the Shape Task and the Conceptual Task in the IS group (N =16). Bilateral ILOTC, aIPS, pIPS, and the frontal regions showed significantly higher-level activation in the shape task than in the conceptual task (vertex-wise $p < 0.001$, cluster-level FWE corrected $p < 0.05$). The ILOTC found here overlapped with the LOtv region in the literature. No significant brain areas showed higher-level activation in the conceptual task than in the shape task (vertex-wise $p < 0.001$, cluster-level FWE corrected $p < 0.05$). However, using a lower threshold (vertex-wise uncorrected $p < 0.05$), we could find the left perisylvian network more activated in the conceptual task than in the shape task.

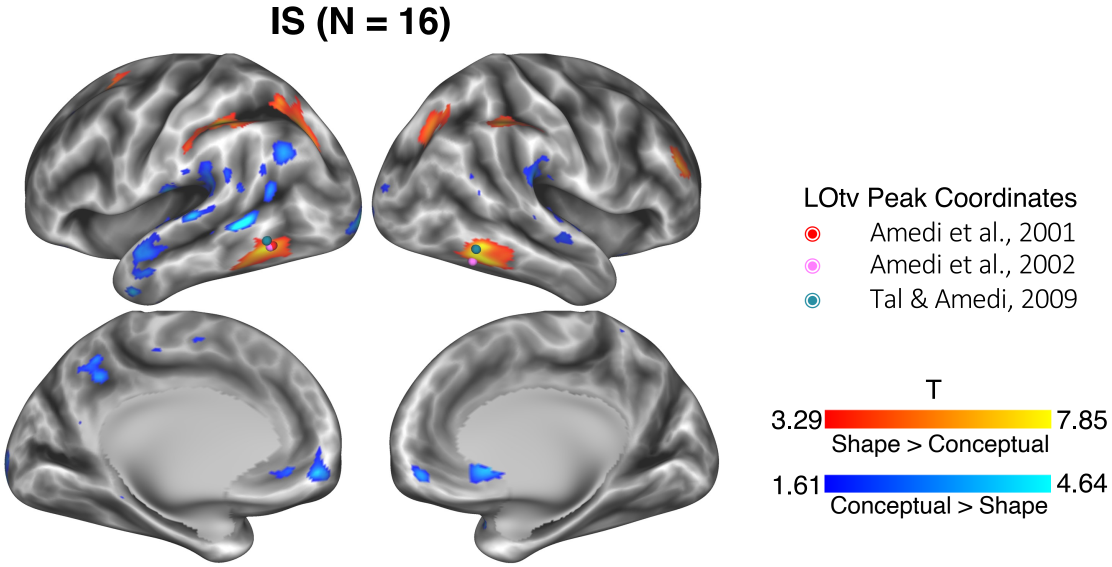

Figure 1. Univariate contrasts between shape and conceptual tasks in the independent sighted (IS) group. The threshold of the Shape > Conceptual contrast: vertex-wise $p < 0.001$, cluster-level FWE corrected $p < 0.05$. The threshold of the Conceptual > Shape contrast: vertex-wise uncorrected $p < 0.05$.

Figure 2 shows the RSA results of bilateral lLOTC in the IS group (N =16). Bilateral lLOTC in the IS group represented shape similarity in both shape task (left lLOTC: $t(15) = 8.476$, $p < 0.001$; right lLOTC: $t(15) = 6.084$, $p < 0.001$) and conceptual task (left lLOTC: $t(15) = 3.553$, $p = 0.003$; right lLOTC: $t(15) = 2.673$, $p = 0.017$). The shape representation was stronger in the shape task than in the conceptual task (left lLOTC: paired $t(15) = 6.622$, $p < 0.001$; right lLOTC: paired $t(15) = 3.937$, $p = 0.001$). We found no significant evidence that bilateral lLOTC represented the conceptual association in either the shape or the conceptual tasks ($t(15) < 1.984$, ps > 0.066).

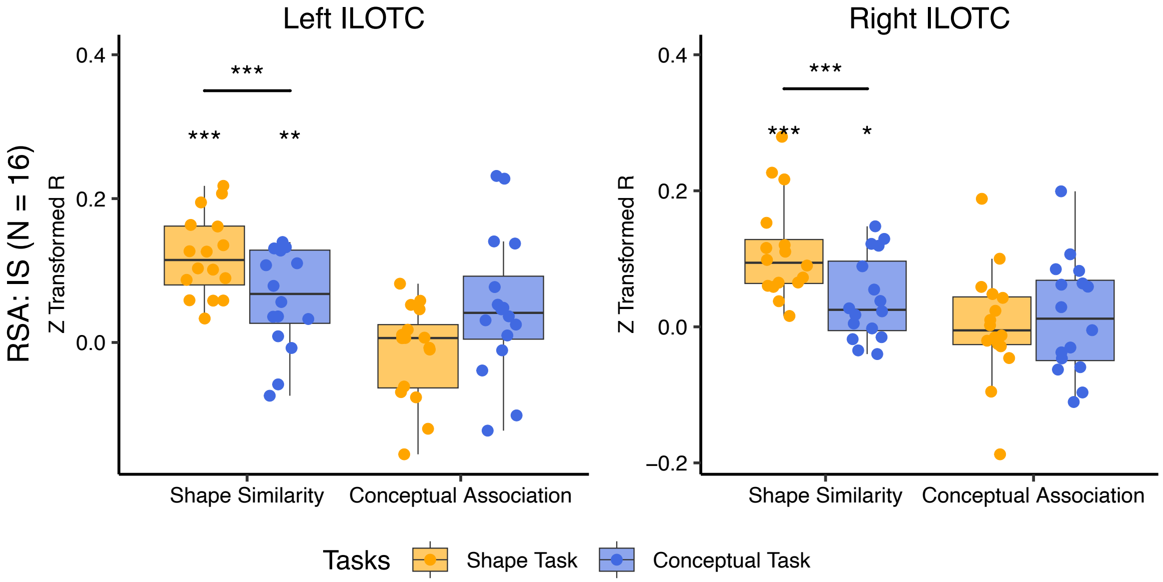

Figure 2. RSA results of bilateral lLOTC in the independent sighted (IS) group. *: $p < 0.05$; **: $p < 0.01$, ***: $p \leq 0.001$.

Q3. Line 492. "similarly" should probably be "similarity"

R3. Thanks for pointing it out. Revised.

Q4. Figure 4D. Why are there two MDS plots? Are they for right and left lLOTC or for a 3-dimensional MDS?

R4. We are sorry for the confusion. They are for left and right lLOTC, not for 3-dimensional MDS. The title of Figure 4A (i.e., "Left lLOTC" and "Right lLOTC") were supposed to be shared by all the subplots in Figure 4. However, the legend below Figure 4C separated Figure 4D from Figures A-C. To avoid this confusion, we have added titles (i.e., "Left lLOTC" and "Right lLOTC") to Figure 4D.

Reference

Andraszewicz, S., Scheibehenne, B., Rieskamp, J., Grasman, R., Verhagen, J., & Wagenmakers, E. J. (2015). An introduction to Bayesian hypothesis testing for management research. *Journal of Management*, 41(2), 521-543.

Barsalou, L. W., Santos, A., Simmons, W. K., & Wilson, C. D. (2008). Language and simulation in conceptual processing. *Symbols, embodiment, and meaning*, 245-283.

Bedny, M. (2017). Evidence from blindness for a cognitively pluripotent cortex. *Trends in cognitive sciences*, 21(9), 637-648.

Bedny, M., & Saxe, R. (2012). Insights into the origins of knowledge from the cognitive neuroscience of blindness. *Cognitive Neuropsychology*, *29*(1-2), 56-84.

Bi, Y. (2021). Dual coding of knowledge in the human brain. *Trends in Cognitive Sciences,* 25(10), 883-895.

Bi, Y., Wang, X., & Caramazza, A. (2016). Object domain and modality in the ventral visual pathway. *Trends in cognitive sciences*, *20*(4), 282-290.

Binkofski, F., Kunesch, E., Classen, J., Seitz, R. J., & Freund, H. J. (2001). Tactile apraxia: unimodal apractic disorder of tactile object exploration associated with parietal lobe lesions. *Brain,* 124(1), 132-144.

Dettmers, C., Liepert, J., Hamzei, F., Binkofski, F., & Weiller, C. (2003). A lesion in the ventrolateral premotor cortex causes difficulties in grasping. *Aktuelle Neurologie*, 30(5), 247-255.

Dove, G. (2009). Beyond perceptual symbols: A call for representational pluralism. *Cognition*, *110*(3), 412-431.

Dove, G. (2011). On the need for embodied and dis-embodied cognition. *Frontiers in Psychology*, *1*, 242.

Fedorenko, E., Behr, M. K., & Kanwisher, N. (2011). Functional specificity for high-level linguistic processing in the human brain. *Proceedings of the National Academy of Sciences*, *108*(39), 16428-16433.

Friederici, A. D. (2011). The brain basis of language processing: from structure to function. *Physiological Reviews*, *91*(4), 1357-1392.

Hömke, L., Amunts, K., Bönig, L., Fretz, C., Binkofski, F., Zilles, K., & Weder, B. (2009). Analysis of lesions in patients with unilateral tactile agnosia using cytoarchitectonic probabilistic maps. *Human Brain Mapping*, *30*(5), 1444-1456.

Kosslyn, S. M., & Thompson, W. L. (2003). When is early visual cortex activated during visual mental imagery? *Psychological bulletin*, *129*(5), 723.

Lane, C., Kanjlia, S., Omaki, A., & Bedny, M. (2015). “Visual” cortex of congenitally blind adults responds to syntactic movement. *Journal of Neuroscience*, *35*(37), 12859-12868.

Mahon, B. Z., & Caramazza, A. (2008). A critical look at the embodied cognition hypothesis and a new proposal for grounding conceptual content. *Journal of physiology-Paris*, *102*(1-3), 59-70.

Miles, J., & Shevlin, M. (2001). *Applying regression and correlation: A guide for students and researchers*. Sage.

Paivio, A. (1990). *Mental representations: A dual coding approach*. Oxford university press.

Pearson, J. (2019). The human imagination: the cognitive neuroscience of visual mental imagery. *Nature reviews neuroscience*, *20*(10), 624-634.

Reilly, J., Peelle, J. E., Garcia, A., & Crutch, S. J. (2016). Linking somatic and symbolic representation in semantic memory: the dynamic multilevel reactivation framework. *Psychonomic bulletin & review*, *23*, 1002-1014.

Ricciardi, E., Bonino, D., Pellegrini, S., & Pietrini, P. (2014). Mind the blind brain to understand the sighted one! Is there a supramodal cortical functional architecture? *Neuroscience & Biobehavioral Reviews*, 41, 64-77.

Rosenke, M., Van den Hurk, J., Margalit, E., Op de Beeck, H. P., Grill-Spector, K., & Weiner, K. S. (2020). Extensive individual differences of category information in ventral temporal cortex in the congenitally blind. *bioRxiv*, 2020-06.

Sen, S., Khalsa, N. N., Tong, N., Ovadia-Caro, S., Wang, X., Bi, Y., & Striem-Amit, E. (2022). The role of visual experience in individual differences of brain connectivity. *Journal of Neuroscience*, 42(25), 5070-5084.

Van Ackeren, M. J., Barbero, F. M., Mattioni, S., Bottini, R., & Collignon, O. (2018). Neuronal populations in the occipital cortex of the blind synchronize to the temporal dynamics of speech. *ELife*, *7*.

van Doorn, J., Aust, F., Haaf, J. M., Stefan, A. M., & Wagenmakers, E. J. (2021). Bayes factors for mixed models. *Computational Brain & Behavior*, 1-13.

Vigliocco, G., Meteyard, L., Andrews, M., & Kousta, S. (2009). Toward a theory of semantic representation. *Language and Cognition*, *1*(2), 219-247.

Winkler, A. M., Ridgway, G. R., Webster, M. A., Smith, S. M., & Nichols, T. E. (2014). Permutation inference for the general linear model. *Neuroimage*, *92*, 381-397.

Xu, Y., He, Y., & Bi, Y. (2017). A tri-network model of human semantic processing. *Frontiers in psychology*, *8*, 1538.

Zwaan, R. A. (2014). Embodiment and language comprehension: Reframing the Discussion. *Trends in cognitive sciences*, *18*(5), 229-234.
